# Supplementary material for: Colour-emotion associations in individuals with red-green colour blindness
Source: PeerJ. 2021 Apr 7;9:e11180. doi: 10.7717/peerj.11180 (PMC8035895; doi:10.7717/peerj.11180)
Supplement: Supplemental Information 1 [file peerj-09-11180-s001.pdf]

Supplemental Material

Supplemental Method Details

Colour vision test scoring and categorisation

Ishihara test. The Ishihara test (Ishihara, 2000) assesses colour confusion along the red-green axis with a series of plates. On each plate, participants see a larger circle filled with smaller circles of different colours. The smaller circles form different numbers or shapes, which participants have to recognise. The plates are presented perpendicular to participants' eyes with an eye-plate distance of about 50 cm distance. The test takes 3-5 minutes to complete.

Each missed or misread number or shape on the first 11 plates is counted as an error. Participants who make four or more errors are considered colour-blind (Thiadens et al., 2013). Participants who make two or three errors are classified as "unsure". Participants who make one or no errors pass the test. Plates 12 to 14 are used to identify the type of colour blindness and we used readings on two out of three plates. Using this criterion, 48 colour-blind participants were categorised as having deutan-like deficiencies. The type of deficiency could not be determined for 16 colour-blind participants, as they either made other types of errors or could not read any number and/or trace any line on two out of three plates. Five colour-blind participants correctly read the plates. None of the non-colour-blind participants made an error on these plates.

Farnsworth D-15 and Lanthony D-15 tests. Farnsworth D-15 test (Farnsworth, 1947) is a colour arrangement test designed to detect colour vision deficiencies. Lanthony D-15 test (Lanthony, 1978a,b) is a desaturated version (Munsell Chroma 2) of the Farnsworth D-15 test. Lanthony D-15 test is more sensitive than Farnsworth D-15 test and can identify individuals with even mild colour blindness.

We used the magnetic version of the Farnsworth D-15 and Lanthony D-15 tests (Good Lite™) to reduce errors due to smudging or physical damage. In this version, the colour samples are presented as disks in a transparent plastic box. The transparent box is placed on a black sheet which acts as background during testing. The disks are moved with a magnetic stick to arrange them according to similarity starting from the reference disk. Each disk is numbered underneath for scoring (not visible to participants). The test takes 3-5 minutes to complete.

Farnsworth D-15 and Lanthony D-15 tests are scored by recording the sequence of disks selected by the participant. Two types of errors occur in D-15 tests. The neighbour errors occur when two neighbouring disks are exchanged. Neighbour errors are counted if the closest neighbour of a disk is exchanged with the second or third closest neighbour. From the fourth closest neighbour onwards, errors are counted as crossing errors (Hovis, Ramaswamy & Anderson, 2004), which are most indicative of colour blindness. The total number of errors is an arithmetic sum of neighbour and crossing errors.

The criteria for colour blindness are based on the number of crossing errors (Farnsworth, 1947; Hovis, Ramaswamy & Anderson, 2004). Participants fail the Farnsworth or Lanthony D-15 test if they make three or more crossing errors. Participants pass the test if they make no crossing errors. Participants who make one or two crossing errors cannot be unequivocally classified as passing or failing the test (classified as “unsure”). Farnsworth D-15 test can differentiate between participants with no colour vision deficiency (“pass”) and participants with strong colour vision deficiency (“fail” or “unsure”). Participants with mild colour vision deficiencies would often pass the Farnsworth D-15 test or be classified as “unsure”. The Lanthony D-15 test is more sensitive and can be used to differentiate between participants with no colour vision deficiency (“pass”) and participants with mild colour vision deficiency (“fail” or “unsure”).

Apparatus

The task was performed on a single Colour Edge CG243W 24.1" Widescreen LCD display, which was linearized with an in-built sequence before each session. We used the Konica Minolta CS-100A chroma meter to measure the parameters of red, green, and blue guns of the monitor. The white point of the monitor was .319, .338, 94.2 in *Commission Internationale d’Eclairage (CIE) xyY* colour space. The gamma curves were estimated from luminance increments of each of the three guns using a standard protocol (Brainard, Pelli & Robson, 2002). The measured primaries in *CIE xyY* of the monitor were Red = (.690, .306, 24.9), Green = (.185, .703, 60), and Blue = (.141, .027, 2.76). We used these measurements to convert colour values from the monitor-independent *CIE* xyY system to the monitor-dependent RGB system we needed to display them on screen. Viewing was unrestrained and the viewing distance was approximately 70 cm.

Supplemental Analyses and Results

Colour vision tests

Scores on all three colour blindness tests were used to evaluate the presence and the degree of colour blindness. Participants who passed two out of three tests were considered to have passed the colour vision tests in general. Participants who passed fewer than two of the three tests (i.e., received scores "fail" or "unsure" on two out of three tests) were considered to have failed the colour vision tests in general (see Table S 5).

We used a 2 x 2 independent-measures MANOVA to test whether colour-blind participants had different colour blindness test scores than non-colour-blind participants, and if there were differences between the two conditions. For these analyses, we included all participants (N = 129, $n_{\text{Colour-blind}}$ = 64, $n_{\text{Non-colour-blind}}$ = 65). The between-subjects independent variables were 1) self-reported colour blindness group (colour-blind vs. non-colour-blind) and 2) condition (colour term vs. colour patch). The dependent variables were 1) number of errors on Ishihara test, 2) number of crossing errors on Farnsworth D-15 test, 3) number of neighbour errors on Farnsworth D-15 test, 4) number of total errors on Farnsworth D-15 test, 5) number of crossing errors on Lanthony D-15 test, 6) number of neighbour errors on Lanthony D-15 test, and 7) number of total errors on Lanthony D-15 test.

The 2 x 2 MANOVA on the number of errors in colour blindness test scores was overall significant; Pillai's Trace value = .89, $F(5, 121) = 198$, $p < .001$, $\eta_p^2 = .891$. The MANOVA revealed a significant main effect of self-reported study groups, Pillai's Trace value = .82, $F(5, 121) = 114$, $p < .001$, $\eta_p^2 = .820$. The main effect of study group was present in all individual scores on the colour vision tests: 1) number of errors on Ishihara test, $F(1, 125) = 323$, $p < .001$, $\eta_p^2 = .721$, 2) number of crossing errors on Farnsworth D-15 test, $F(1, 125) = 78.7$, $p < .001$, $\eta_p^2 = .386$, 3) number of neighbour errors on Farnsworth D-15 test, $F(1, 125) = 21.3$, $p < .001$, $\eta_p^2 = .146$, 4) number of total errors on Farnsworth D-15 test, $F(1, 125) = 99.2$, $p < .001$, $\eta_p^2 = .443$, 5) number of crossing errors on Lanthony D-15 test, $F(1, 125) = 263$, $p < .001$, $\eta_p^2 = .678$, 6) number of neighbour errors on Lanthony D-15 test, $F(1, 125) = 24.6$, $p < .001$, $\eta_p^2 = .165$, and 7) number of total errors on Lanthony D-15 test, $F(1, 125) = 340$ $p < .001$, $\eta_p^2 = .731$. In all tests, self-reported colour-blind participants made significantly more errors than self-reported non-colour-blind participants (see Table S 5 for mean scores of each test).

As there was no main effect of condition, Pillai's Trace value = .02, $F(5, 121) = 0.35$, $p = .83$, $\eta_p^2 = .017$, we concluded that participant allocation to the conditions was random. The interaction between study group and condition was not significant either, Pillai's Trace value = .05, $F(5, 121) = 1.24$, $p = .29$, $\eta_p^2 = .049$, reinforcing the same conclusion.

Based on the re-categorisation, Table S 4 reports the number of participants included in re-categorised study groups. The same table also reports mean colour blindness index scores of both study groups in

both conditions. Clearly, colour-blind participants had significantly higher colour blindness index scores than non-colour-blind participants, confirming their colour vision deficiency.

Emotion intensity

Group-level analysis. The current study compares associations of colour blind and non-colour-blind participants. Consequently, we present emotion intensities split by colour for colour-blind participants (Table S 6) and non-colour-blind participants (Table S 7). We compared emotion associations between colour terms and colour patches. These comparisons revealed that colour-blind participants associated emotion concepts of higher intensity with *red*, *orange*, *yellow*, *pink*, *white*, and *black* as a term than as a patch (all p_{FDR} < .046). Similarly, non-colour-blind participants associated emotion concepts of higher intensity with *red* as a term than as a patch (p_{FDR} = .004). Non-colour-blind participants tended to associate more intense emotion concepts with *white* and *black* as a term than as a patch, which was nearly significant (both p_{FDR} = .051). Additionally, we present a comparison between colour-blind and non-colour-blind participants for emotion intensities split by colour. We present them separately for colour terms (Table S 8) and colour patches (Table S 9).

Individual-level analysis. We tested whether *emotion intensity* depended on the colour blindness index by fitting a linear regression model. The predictor variable was the colour blindness index and the outcome variable was average emotion intensity (across all colours and emotions). We further ran a series of analogous linear regression models per colour to test for the same dependence in each colour. We had to run 12 models per colour and not a single model with all the colours together due to missing data (see *Emotion intensity*). Again, all comparisons were FDR corrected (Benjamini & Hochberg, 1995). The linear regression model was not significant overall, $F(1, 127) = 1.08$, $p = .305$, *adjusted* $R^2 < .001$, indicating that the colour blindness index was not a significant predictor of average emotion intensity. Similarly, the colour blindness index was not a significant predictor of emotion intensity of any of the 12 colours, *p's* ≥ 0.055 (before correction).

Emotion dimensions

We derived emotion dimensions associated with colours from the number of emotion concepts associated with each colour. For valence, we counted how many positive and negative emotion concepts each participant associated with each colour. For arousal, we counted how many high arousal and low arousal emotion concepts each participant associated with each colour. For power, we counted how many high power and low power emotion concepts each participant associated with each colour (Table S 2). The more emotions participants chose, the broader and less specific their

colour-emotion associations were. The number of associated emotions varied from 0 to 10 for each level of valence (positive vs. negative), arousal (high vs. low arousal), and power (high vs. low power).

To compare emotion dimension associations between colour-blind and non-colour-blind participants, we conducted a mixed-design 2 x 2 x 2 x 12 multivariate analysis of variance (MANOVA) model. The three dependent variables were valence, arousal, and power. The independent variables were i) level of emotion dimensions (2 levels, positive – negative, high arousal – low arousal, or high power – low power); ii) study groups (2 levels, colour-blind or non-colour-blind); iii) conditions (2 levels, terms or patches); and iv) colour (12 levels, see *Colour Stimuli*). The interactions of interest were followed up with individual ANOVAs on each dependent variable and t-tests where appropriate. The main effects and interactions that did not concern study group were not further interpreted but could be visually deduced from Figure S 2. as well as inspected in the raw data.

A mixed-design MANOVA estimating the number of associated emotion concepts was overall significant; Pillai's Trace value = .70, $F(1, 131) = 230.6$, $p < .001$, $\eta_p^2 = .695$. However, the main effect of study group was not significant, Pillai's Trace value = .002, $F(1, 101) = 0.23$, $p = .630$, $\eta_p^2 = .002$, suggesting that colour-blind and non-colour-blind participants associated the same number of emotion concepts with all colours on average. The main effect of condition was not significant either, Pillai's Trace value = .000, $F(1, 101) = 0.34$, $p = .854$, $\eta_p^2 = .000$, suggesting that the same number of emotion concepts, on average, was associated with terms and patches. In contrast, the main effect of colour was significant, Pillai's Trace value = .50, $F(11, 91) = 8.32$, $p < .001$, $\eta_p^2 = .502$. The main effect of levels of emotion dimensions was also significant, Pillai's Trace value = .52, $F(3, 99) = 35.2$, $p < .001$, $\eta_p^2 = .516$. This result was further analysed for valence, arousal, and power separately.

The two-way interactions of interest were not significant. These interactions were between 1) study group and levels of emotion dimensions, Pillai's Trace value = .02, $F(3, 99) = 0.73$, $p = .539$, $\eta_p^2 = .022$, 2) study group and colour, Pillai's Trace value = .15, $F(11, 91) = 1.40$, $p = .188$, $\eta_p^2 = .145$, and 3) study group and condition, Pillai's Trace value = .02, $F(1, 101) = 1.79$, $p = .184$, $\eta_p^2 = .017$. Most of the three-way interactions of interest were also not significant. These interactions were between 1) study group, levels of emotion dimensions, and condition, Pillai's Trace value = .01, $F(3, 99) = 0.35$, $p = .788$, $\eta_p^2 = .011$, and 2) study group, colour, and condition, Pillai's Trace value = .05, $F(11, 91) = 0.46$, $p < .001$, $\eta_p^2 = .053$. The only significant three-way interaction of interest was between study group, levels of emotion dimensions, and colour, Pillai's Trace value = .44, $F(33, 69) = 1.63$, $p = .045$, $\eta_p^2 = .438$. This interaction was further analysed for valence, arousal, and power separately. The four-way interaction

between study group, levels of emotion dimensions, colour, and condition was not significant, Pillai's Trace value = .42, $F(33, 69) = 1.53$, $p = .070$, $\eta_p^2 = .422$.

In contrast to the interactions of interest, the two-way and three-way interactions of lower interest were significant. These interactions were between 1) levels of emotion dimensions and condition, Pillai's Trace value = .10, $F(3, 99) = 3.83$, $p = .012$, $\eta_p^2 = .104$, 2) colour and condition, Pillai's Trace value = .27, $F(11, 91) = 3.00$, $p = .002$, $\eta_p^2 = .266$, 3) levels of emotion dimensions and colour, Pillai's Trace value = .871, $F(33, 69) = 14.06$, $p < .001$, $\eta_p^2 = .871$, and 4) levels of emotion dimensions, colour, and condition, Pillai's Trace value = .51, $F(33, 69) = 2.21$, $p = .003$, $\eta_p^2 = .514$. We did not further interpret these interactions, as they were not of interest to the current study. For visual representation of all results, see Figure S 2.

Valence. Following up the results of the mixed-design MANOVA with a mixed-design ANOVA on valence, there was the main effect of valence level, $F(1, 101) = 107$, $p < .001$, $\eta_p^2 = .515$, indicating a positivity bias. Participants overall associated more positive ($M = 2.43$, 95% $CI = [2.13, 2.74]$) than negative ($M = 1.47$, 95% $CI = [1.24, 1.71]$) emotion concepts with colours. This main effect was qualified by the interactions between valence level and colour, $F(11, 1111) = 58.6$, $p < .001$, $\eta_p^2 = .367$, valence level, colour, and condition, $F(11, 1111) = 2.21$, $p = .012$, $\eta_p^2 = .021$, and, most pertinent, valence level, colour, and study group, $F(11, 1111) = 2.64$, $p = .002$, $\eta_p^2 = .025$. We interpret the latter interaction below while the meaning of the two former interactions can be visually deduced from Figure S 2.

To break-down the interaction between valence level, colour, and study group, we performed 12 2 x 2 x 2 ANOVA models, one per colour, with valence level (positive vs. negative) and study group (colour-blind vs. non-colour-blind) as independent variables. The main effect of study group was only significant for *red*, $F(1,103) = 4.47$, $p = .037$, $\eta_p^2 = .042$. That is, colour-blind participants associated fewer emotion concepts with *red* than non-colour-blind participants, irrespective of whether *red* was a term or a patch. The main effects of study group for other colours were not significant ($ps \geq .328$). The interaction between study group and condition was significant for *turquoise*, $F(1,103) = 7.98$, $p = .006$, $\eta_p^2 = .072$, *purple*, $F(1,103) = 8.40$, $p = .005$, $\eta_p^2 = .075$, and *pink*, $F(1,103) = 4.00$, $p = .048$, $\eta_p^2 = .037$. Further series of paired-samples t-tests showed that colour-blind participants associated more positive than negative emotion concepts with *turquoise* ($p_{FDR} < .001$) and *pink* ($p_{FDR} < .001$) but not with *purple* ($p_{FDR} = .826$). In contrast, non-colour-blind participants associated more positive than negative emotion concepts with *turquoise* ($p_{FDR} < .001$), *pink* ($p_{FDR} < .001$), and *purple* ($p_{FDR} < .001$).

Thus, at the core of this interaction was valence of *purple* – non-colour-blind participants evaluated *purple* as a positive colour while colour-blind participants evaluated *purple* as an ambivalent colour.

Arousal. Following up on the results of the mixed-design MANOVA with a mixed-design ANOVA on arousal, there was no main effect of arousal level, $F(1, 101) = 1.63$, $p = .204$, $\eta_p^2 = .016$. There were significant two-way and three-way interactions but none of them included study group. These interactions were between 1) arousal level and condition, $F(1, 101) = 11.3$, $p = .001$, $\eta_p^2 = .101$, 2) arousal level and colour, $F(11, 1111) = 45.0$, $p < .001$, $\eta_p^2 = .308$, and 3) arousal level, colour, and condition, $F(11, 1111) = 4.18$, $p < .001$, $\eta_p^2 = .040$. Since these interactions were not of interest, their meaning can be visually deduced from Figure S 2. A&C.

Power. Following up on the results of the mixed-design MANOVA with a mixed-design ANOVA on power, there was the main effect of power level, $F(1, 101) = 4.23$, $p = .042$, $\eta_p^2 = .040$. Participants overall associated slightly more high power ($M = 2.00$, *95% CI* = [1.74, 2.27]) than lower power ($M = 1.90$, *95% CI* = [1.65, 2.16]) emotion concepts with colours. The only significant interaction was between power level and colour, $F(11, 1111) = 19.1$, $p < .001$, $\eta_p^2 = .159$. Since it was not of interest, we did not further interpret this interaction and its meaning can be visually deduced from Figure S 2. B&D.

Colour naming

We compared colour naming between colour-blind and non-colour-blind participants in the patches condition. We present the average likelihood mean values that each colour patch was named using each colour name in Table S 14 for colour-blind and Table S 15 for non-colour-blind participants. The responses of colour-blind participants were highly correlated to the responses of non-colour-blind participants, $r = .943$, $p < .001$.

References appearing in this section

Benjamini Y, Hochberg Y. 1995. Controlling the false discovery rate: A practical and powerful approach to multiple testing. *Journal of the Royal Statistical Society. Series B (Methodological)* 57:289–300. DOI: 10.2307/2346101.

Brainard DH, Pelli DG, Robson T. 2002. Display characterization. In: Hornak J ed. *Encyclopedia of imaging science and technology*. Hoboken, NJ: John Wiley & Sons, Inc., 172–188. DOI: 10.1002/0471443395.img011.

Farnsworth D. 1947. *The Farnsworth dichotomous test for color blindness - panel D15*. New York, NY: The Psychological Corp.

Fontaine JRJ. 2013. Dimensional, basic emotion, and componential approaches to meaning in psychological emotion research. In: *Components of emotional meaning: A sourcebook*. Oxford:

Oxford University Press, 31–45. DOI: 10.1093/acprof:oso/9780199592746.003.0003.

Forbes I. 1979. The terms brun and marron in modern standard French. *Journal of Linguistics* 15:295–305. DOI: 10.1017/S0022226700016406.

Hovis JK, Ramaswamy S, Anderson M. 2004. Repeatability indices for the Farnsworth D-15 test. *Visual Neuroscience* 21:449–453. DOI: 10.1017/S0952523804213402.

Ishihara S. 2000. *The series of plates designed as a test for color deficiency. Concise edition*. Tokyo: Kanehara & Co (Original work published 1917).

Jonauskaite D, Parraga CA, Quiblier M, Mohr C. 2020. Feeling blue or seeing red? Similar patterns of emotion associations with colour patches and colour terms. *i-Perception* 11:1–24. DOI: 10.1177/2041669520902484.

Lanthony P. 1978a. The new color test. *Documenta Ophthalmologica* 46:191–199. DOI: 10.1007/BF00174108.

Lanthony P. 1978b. The desaturated panel D-15. *Documenta Ophthalmologica* 46:185–189. DOI: 10.1007/BF00174107.

Scherer KR. 2005. What are emotions? And how can they be measured? *Social Science Information* 44:695–729. DOI: 10.1177/0539018405058216.

Scherer KR, Shuman V, Fontaine JRJ, Soriano C. 2013. The GRID meets the Wheel: Assessing emotional feeling via self-report. In: Fontaine JRJ, Scherer KR, Soriano C eds. *Components of emotional meaning: A sourcebook*. Oxford: Oxford University Press, 281–298. DOI: 10.13140/RG.2.1.2694.6406.

Soriano C, Fontaine JRJ, Scherer KR, Akırmak GA, Alarcón P, Alonso-Arbiol I, Bellelli G, Pérez-Aranibar CC, Eid M, Ellsworth P, Galati D, Hareli S, Hess U, Ishii K, Jonker C, Lewandowska-Tomaszczyk B, Meiring D, Mortillaro M, Niiya Y, Ogarkova A, Panasenko N, Ping H, Protopapas A, Realo A, Ricci-Bitti PE, Shen Y-L, Sheu C-F, Siiroinen M, Sunar D, Tissari H, Tong EMW, van Osch Y, Wong S, Yeung DY, Zitouni A. 2013. Cross-cultural data collection with the GRID instrument. In: *Components of emotional meaning: A sourcebook*. Oxford: Oxford University Press, 98–105. DOI: 10.1093/acprof:oso/9780199592746.003.0007.

Spence NCW. 1989. The Linguistic Field of Colour Terms in French. *Zeitschrift fur Romanische Philologie* 105:472–497. DOI: 10.1515/zrph.1989.105.5-6.472.

Thiadens AAHJ, Hoyng CB, Polling JR, Bernaerts-Biskop R, van den Born LI, Klaver CCW. 2013. Accuracy of four commonly used color vision tests in the identification of cone disorders. *Ophthalmic Epidemiology* 20:114–122. DOI: 10.3109/09286586.2012.759596.

Supplemental Figures

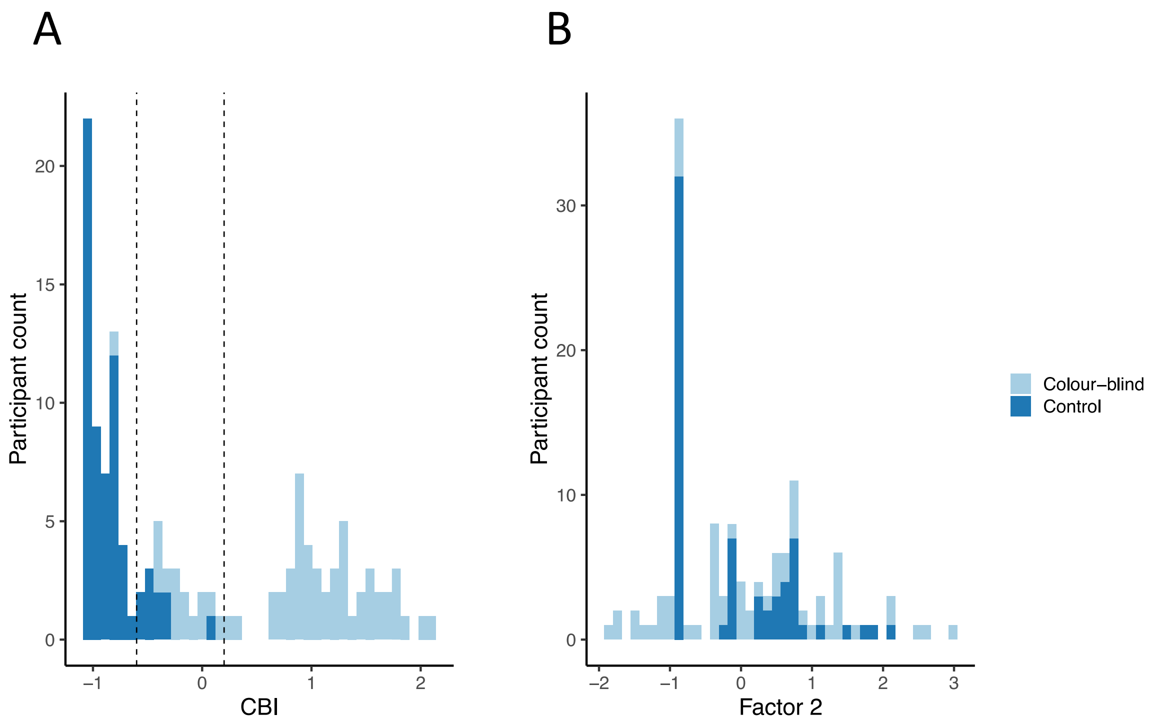

Figure S 1. Solutions of the principal component analysis, separated by self-identified study group. (A) Colour blindness index (CBI), dotted lines indicate the separation of participants into re-categorised colour-blind participants (to the right of the rightward line at 0.2) and re-categorised non-colour-blind participants (i.e., control, to the left of the leftward line at -0.6). (B) Factor 2 of the factor analysis, which clearly does not separate colour-blind and non-colour-blind participants. Thus, the latter measure was not considered for our further analyses. See Table S 3 for factor loadings.

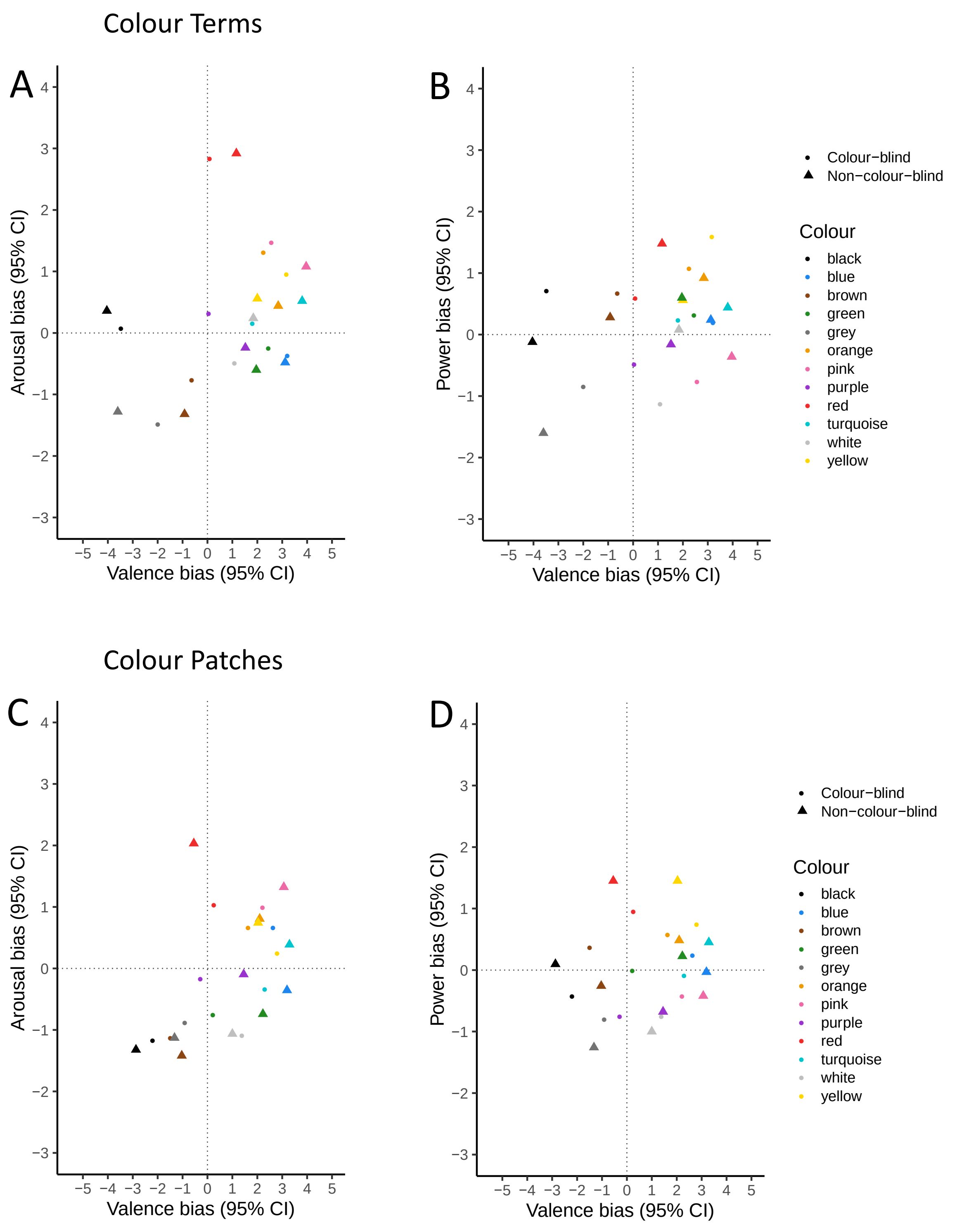

Figure S 2. Valence, arousal, and power biases of colour, separated by colour-blind participants (circles) and non-colour-blind participants (triangles). (A & C) Colour terms (A) or colour patches (C) positioned on the valence x arousal space. Valence bias was calculated by subtracting the number of negative emotion concepts from the number of positive emotion concepts associated with each colour (positive – negative); higher values indicate a more positive evaluation. Arousal bias calculated by subtracting the number of low arousal emotion concepts from the number of high arousal emotion concepts associated with each colour (high arousal – low arousal); higher values indicate a more

arousing evaluation. (B & D) Colour terms (B) or colour patches (D) positioned on the valence x power space. Power bias calculated by subtracting weak emotion concepts from strong emotion concepts associated with each colour (strong – weak); higher values indicate a more empowering evaluation. (A & B) Error bars indicate 95% confidence intervals (*CI*) of the mean. Dotted lines indicate the separation between positive-negative, high arousal-low arousal, and strong-weak emotion concepts. Colours are for visualisation purposes only.

Supplemental Tables

Table S 1. Colour and emotion terms in English and French. The validated French version of emotion terms taken from http://www.affective-sciences.org/gew. French has two basic terms for the "brown" category – *brun* and *marron* (Forbes, 1979). We chose *brun* since it has the least contextually restricted meaning (Spence, 1989). However, we hypothesise that *marron* would result in very similar affective associations to *brun,* since both *brun* and *marron* map to comparable perceptual colours (Spence, 1989).

English	French
Red	Rouge
Orange	Orange
Yellow	Jaune
Green	Vert
Turquoise	Turquoise
Blue	Bleu
Purple	Violet
Pink	Rose
Brown	Brun
White	Blanc
Grey	Gris
Black	Noir
Interest	Intérêt
Amusement	Amusement
Pride	Fierté
Joy	Joie
Pleasure	Plaisir
Contentment	Contentement
Admiration	Admiration
Love	Amour
Relief	Soulagement
Compassion	Compassion
Sadness	Tristesse
Guilt	Culpabilité
Regret	Regret
Shame	Honte
Disappointment	Déception
Fear	Peur
Disgust	Dégoût
Contempt	Mépris
Hate	Haine
Anger	Colère

Forbes, I. (1979). The terms brun and marron in modern standard French. *Journal of Linguistics*, *15*(2), 295–305. https://doi.org/10.1017/S0022226700016406

Table S 2. The 20 emotions used in the Geneva Emotion Wheel. We categorised them on three emotion dimensions of valence, arousal, and power (Scherer, 2005; Scherer et al., 2013; Fontaine, 2013; Soriano et al., 2013; Jonauskaite et al., 2020)

Emotion	Valence	Arousal	Power
Interest	Positive	Low	Strong
Amusement	Positive	High	Strong
Pride	Positive	Low	Strong
Joy	Positive	High	Strong
Pleasure	Positive	High	Strong
Contentment	Positive	Low	Weak
Admiration	Positive	High	Weak
Love	Positive	High	Weak
Relief	Positive	Low	Weak
Compassion	Positive	Low	Weak
Sadness	Negative	Low	Weak
Guilt	Negative	High	Weak
Regret	Negative	Low	Weak
Shame	Negative	High	Weak
Disappointment	Negative	Low	Weak
Fear	Negative	High	Strong
Disgust	Negative	Low	Strong
Contempt	Negative	Low	Strong
Hate	Negative	High	Strong
Anger	Negative	High	Strong

Table S 3. Item-loadings on the two factors. The first factor was identified as Colour Blindness Index while the second factor was difficult to interpret and was left unnamed.

Item	Colour blindness index (Factor 1)	Factor 2
Ishihara errors	.900	-.082
Farnsworth D-15 crossing errors	.846	-.326
Farnsworth D-15 neighbour errors	.533	.483
Lanthony D-15 crossing errors	.948	-.152
Lanthony D-15 neighbour errors	.283	.835

Table S 4. Participants re-categorised as colour-blind or non-colour-blind based on their Colour Blindness Index score. These participants were included in group-level analyses. Superscript letters ([a,b]) indicate significant differences at $p < .001$.

		N	Colour blindness index	
			Mean	*SD*
Colour terms condition	Colour blind	25	1.20^{a}	0.43
	Non-colour-blind	25	-0.93^{a}	0.10
Colour patches condition	Colour blind	24	1.17^{b}	0.41
	Non-colour-blind	31	-0.92^{b}	0.11

Table S 5. Colour blindness test results of the colour-blind and non-colour-blind participants. Participants passed colour blindness tests in general if they passed at least two out of three colour blindness tests (*Final decision*). Passing criteria for each individual test and how errors were counted appear in text.

		**Ishihara test**				**Farnsworth D-15 test**						**Lanthony D-15 test**						**Final decision**	
		Pass (*n*; %)	Fail (*n*; %)	Unsure (*n*; %)	No. errors (*M*, *SD*, *range*)	Pass (*n*; %)	Fail (*n*; %)	Unsure (*n*; %)	No. crossing errors (*M*, *SD*, *range*)	No. neighbour errors (*M*, *SD*, *range*)	No. total errors (*M*, *SD*, *range*)	Pass (*n*; %)	Fail (*n*; %)	Unsure (*n*; %)	No. crossing errors (*M*, *SD*, *range*)	No. neighbour errors (*M*, *SD*, *range*)	No. total errors (*M*, *SD*, *range*)	Pass (*n*; %)	Fail (*n*; %)
**Colour terms condition**	**Colour-blind**	0 (0.0)	27 (90.0)	3 (10.0)	7.3 (2.8), 2-11	8 (26.7)	19 (63.3)	3 (10.0)	4.9 (3.7), 0-11	1.5 (1.6), 0-6	6.4 (4.0), 0-12	2 (6.7)	27 (90.0)	1 (3.3)	6.23 (2.7), 0-10	3.0 (1.7), 0-6	9.2 (1.8), 4-12	2 (6.7)	28 (93.3)
	**Non colour-blind**	31 (100)	0 (0.0)	0 (0.0)	0.2 (0.4), 0-1	31 (100)	0 (0.0)	0 (0.0)	0.0 (0.0), 0	0.6 (1.4), 0-5	0.6 (1.4), 0-5	26 (83.9)	2 (6.5)	3 (9.7)	0.3 (0.8), 0-3	1.7 (1.9), 0-6	2.0 (2.3), 0-7	31 (100)	0 (0.0)
**Colour patches condition**	**Colour-blind**	0 (0.0)	26 (76.5)	8 (23.5)	6.2 (2.9), 2-10	14 (41.2)	17 (50.0)	3 (8.8)	3.7 (3.9), 0-12	1.7 (1.8), 0-7	5.4 (4.3), 0-14	1 (2.9)	27 (79.4)	6 (17.6)	5.6 (2.8), 0-10	3.3 (2.0), 0-8	8.9 (2.8), 0-13	1 (2.9)	33 (97.1)
	**Non colour-blind**	32 (94.1)	0 (0.0)	2 (5.9)	0.4 (0.6), 0-2	33 (97.1)	1 (2.9)	0 (0.0)	0.1 (0.5), 0-3	0.3 (0.5), 0-2	0.4 (1.0), 0-5	31 (91.2)	0 (0.0)	3 (8.8)	0.1 (0.4), 0-2	1.4 (1.8), 0-7	1.5 (1.9), 0-7	34 (100)	0 (0.0)

Table S 6. Descriptive values of the intensity of the associated emotion concepts with colour terms and colour patches by colour-blind participants. Significant differences, after the FDR correction, in emotion intensity between the two conditions are flagged as * $p_{FDR} < .050$, ** $p_{FDR} < .010$.

	Colour terms			Colour patches					
Colour blind	*M*	95% *CI*	Range	*M*	95% *CI*	Range	*N*	*t*-value	Cohen's *d*
Red	4.17	[3.88,4.46]	3.00-5.00	3.18	[2.73,3.62]	1.33-4.67	47	3.96**	1.16
Orange	3.73	[3.40,4.07]	2.00-5.00	2.87	[2.46,3.27]	1.00-4.67	47	3.41**	1.00
Yellow	3.99	[3.72,4.27]	2.26-5.00	3.39	[2.96,3.81]	1.67-5.00	49	2.50*	0.71
Green	3.76	[3.43,4.08]	1.75-5.00	3.29	[2.94,3.64]	1.50-5.00	45	2.04	0.61
Turquoise	3.43	[3.14,3.73]	2.50-5.00	3.25	[2.83,3.66]	1.33-4.67	43	0.75	0.23
Blue	3.80	[3.54,4.06]	2.67-4.50	3.51	[3.14,3.87]	1.30-5.00	48	1.36	0.39
Purple	3.44	[3.03,3.84]	2.00-5.00	2.95	[2.45,3.44]	1.60-5.00	44	1.61	0.48
Pink	3.61	[3.27,3.95]	2.00-5.00	2.85	[2.34,3.36]	1.00-4.67	45	2.61*	0.78
Brown	3.17	[2.68,3.66]	1.71-5.00	3.13	[2.71,3.55]	1.25-5.00	42	0.13	0.04
White	3.78	[3.27,4.29]	2.00-5.00	3.10	[2.76,3.45]	1.75-5.00	43	2.33*	0.71
Grey	3.42	[3.08,3.77]	1.92-5.00	2.92	[2.48,3.36]	1.63-5.00	44	1.91	0.58
Black	3.87	[3.49,4.25]	1.80-5.00	2.99	[2.53,3.46]	1.00-5.00	46	3.03*	0.89
Overall	3.71	[3.52,3.91]	2.68-4.64	3.16	[2.86,3.45]	1.78-4.54	49	3.26**	0.93

Table S 7. Descriptive values of the intensity of the associated emotion concepts with colour terms and colour patches by non-colour-blind participants. Significant differences, after the FDR correction, in emotion intensity between the two conditions are flagged as *** $p_{FDR} < .001$, ∽ $p_{FDR} = .051$

Non-colour-blind	Colour terms			Colour patches					
	M	95% *CI*	Range	*M*	95% *CI*	Range	*N*	*t*-value	Cohen's *d*
Red	3.95	[3.65,4.25]	2.69-5.00	3.16	[2.87,3.45]	1.11-5.00	56	3.87***	1.04
Orange	3.19	[2.87,3.51]	2.00-4.50	3.10	[2.75,3.45]	1.00-5.00	53	0.37	0.10
Yellow	3.46	[3.14,3.78]	2.00-4.50	3.40	[2.92,3.87]	1.00-5.00	52	0.22	0.06
Green	3.38	[3.02,3.74]	1.50-5.00	2.94	[2.66,3.22]	1.67-5.00	56	2.02	0.54
Turquoise	3.43	[3.03,3.82]	1.00-5.00	3.27	[2.91,3.63]	1.00-5.00	54	0.59	0.16
Blue	3.49	[3.14,3.84]	2.00-4.67	3.02	[2.68,3.37]	1.25-5.00	56	1.93	0.52
Purple	3.02	[2.65,3.38]	1.43-4.67	3.16	[2.77,3.54]	1.33-5.00	54	-0.53	0.15
Pink	3.41	[3.05,3.77]	2.00-5.00	3.04	[2.68,3.39]	1.00-5.00	54	1.50	0.41
Brown	2.73	[2.29,3.17]	1.00-4.33	2.85	[2.47,3.23]	1.00-4.50	50	-0.42	0.12
White	3.91	[3.42,4.40]	2.00-5.00	3.03	[2.56,3.50]	1.00-5.00	47	2.68∽	0.78
Grey	3.47	[3.09,3.85]	1.00-5.00	2.94	[2.50,3.38]	1.00-5.00	50	1.84	0.52
Black	3.48	[3.14,3.82]	2.13-5.00	2.81	[2.44,3.19]	1.00-5.00	55	2.61∽	0.71
Overall	3.45	[3.24,3.67]	2.50-4.26	3.09	[2.82,3.35]	1.64-4.65	56	2.15	0.58

Table S 8. Descriptive values of the intensity of the associated emotion concepts by colour-blind and non-colour-blind participants with colour terms. Significant differences, after the FDR correction, in emotion intensity between the study groups are flagged as * $p_{FDR} < .050$, ** $p_{FDR} < .010$, *** $p_{FDR} < .001$, yet, no comparison was significant.

	Colour blind			Non-colour-blind					
Colour terms	*M*	95% *CI*	Range	*M*	95% *CI*	Range	*N*	*t*-value	Cohen's *d*
Red	4.17	[3.88,4.46]	3.00-5.00	3.95	[3.65,4.25]	2.69-5.00	50	1.09	0.31
Orange	3.73	[3.40,4.07]	2.00-5.00	3.19	[2.87,3.51]	2.00-4.50	47	2.43	0.71
Yellow	3.99	[3.72,4.27]	2.26-5.00	3.46	[3.14,3.78]	2.00-4.50	49	2.63	0.75
Green	3.76	[3.43,4.08]	1.75-5.00	3.38	[3.02,3.74]	1.50-5.00	47	1.59	0.46
Turquoise	3.43	[3.14,3.73]	2.50-5.00	3.43	[3.03,3.82]	1.00-5.00	44	0.02	0.01
Blue	3.80	[3.54,4.06]	2.67-4.50	3.49	[3.14,3.84]	2.00-4.67	49	1.46	0.42
Purple	3.44	[3.03,3.84]	2.00-5.00	3.02	[2.65,3.38]	1.43-4.67	46	1.61	0.47
Pink	3.61	[3.27,3.95]	2.00-5.00	3.41	[3.05,3.77]	2.00-5.00	48	0.85	0.25
Brown	3.17	[2.68,3.66]	1.71-5.00	2.73	[2.29,3.17]	1.00-4.33	40	1.41	0.45
White	3.78	[3.27,4.29]	2.00-5.00	3.91	[3.42,4.40]	2.00-5.00	44	-0.38	0.11
Grey	3.42	[3.08,3.77]	1.92-5.00	3.47	[3.09,3.85]	1.00-5.00	47	-0.19	0.06
Black	3.87	[3.49,4.25]	1.80-5.00	3.48	[3.14,3.82]	2.13-5.00	48	1.57	0.45
Overall	3.71	[3.52,3.91]	2.68-4.64	3.45	[3.24,3.67]	2.50-4.26	50	1.83	0.52

Table S 9. Descriptive values of the intensity of the associated emotion concepts by colour-blind and non-colour-blind participants with colour patches. Significant differences, after the FDR correction, in emotion intensity between the study groups are flagged as * $p_{FDR} < .050$, ** $p_{FDR} < .010$, *** $p_{FDR} < .001$, yet, no comparison was significant.

	Colour-blind			Non-colour-blind					
Colour patches	*M*	95% *CI*	Range	*M*	95% *CI*	Range	*N*	*t*-value	Cohen's *d*
Red	3.18	[2.73,3.62]	1.33-4.67	3.16	[2.87,3.45]	1.11-5.00	53	0.08	0.02
Orange	2.87	[2.46,3.27]	1.00-4.67	3.10	[2.75,3.45]	1.00-5.00	53	-0.89	0.25
Yellow	3.39	[2.96,3.81]	1.67-5.00	3.40	[2.92,3.87]	1.00-5.00	52	-0.03	0.01
Green	3.29	[2.94,3.64]	1.50-5.00	2.94	[2.66,3.22]	1.67-5.00	54	1.61	0.44
Turquoise	3.25	[2.83,3.66]	1.33-4.67	3.27	[2.91,3.63]	1.00-5.00	53	-0.11	0.03
Blue	3.51	[3.14,3.87]	1.30-5.00	3.02	[2.68,3.37]	1.25-5.00	55	1.95	0.53
Purple	2.95	[2.45,3.44]	1.60-5.00	3.16	[2.77,3.54]	1.33-5.00	52	-0.71	0.20
Pink	2.85	[2.34,3.36]	1.00-4.67	3.04	[2.68,3.39]	1.00-5.00	51	-0.64	0.18
Brown	3.13	[2.71,3.55]	1.25-5.00	2.85	[2.47,3.23]	1.00-4.50	52	1.03	0.29
White	3.10	[2.76,3.45]	1.75-5.00	3.03	[2.56,3.50]	1.00-5.00	46	0.25	0.07
Grey	2.92	[2.48,3.36]	1.63-5.00	2.94	[2.50,3.38]	1.00-5.00	47	-0.07	0.02
Black	2.99	[2.53,3.46]	1.00-5.00	2.81	[2.44,3.19]	1.00-5.00	53	0.62	0.17
Overall	3.16	[2.86,3.45]	1.78-4.54	3.09	[2.82,3.35]	1.64-4.65	55	0.36	0.10

Table S 10. Colour-emotion association matrix with the proportion of participants who associate given colours with given emotions. These proportions were derived from colour-emotion associations of colour-blind participants with colour terms.

	Red	Orange	Yellow	Green	Turquoise	Blue	Purple	Pink	Brown	Grey	White	Black
Interest	0.00	0.08	0.24	0.32	0.24	0.48	0.12	0.04	0.04	0.16	0.12	0.20
Amusement	0.12	0.44	0.48	0.40	0.36	0.36	0.24	0.40	0.00	0.08	0.00	0.00
Pride	0.12	0.16	0.44	0.12	0.16	0.24	0.08	0.08	0.16	0.08	0.12	0.16
Joy	0.20	0.64	0.76	0.44	0.28	0.36	0.24	0.32	0.04	0.04	0.24	0.04
Pleasure	0.40	0.52	0.72	0.32	0.44	0.44	0.32	0.40	0.08	0.04	0.20	0.04
Contentment	0.08	0.28	0.32	0.32	0.20	0.40	0.12	0.12	0.08	0.08	0.36	0.00
Admiration	0.28	0.24	0.32	0.16	0.16	0.28	0.16	0.36	0.04	0.08	0.24	0.04
Love	0.72	0.16	0.16	0.20	0.12	0.20	0.12	0.68	0.04	0.04	0.12	0.00
Relief	0.00	0.12	0.24	0.44	0.36	0.44	0.12	0.20	0.08	0.08	0.40	0.00
Compassion	0.20	0.16	0.20	0.28	0.16	0.24	0.12	0.44	0.00	0.20	0.28	0.00
Sadness	0.00	0.08	0.04	0.08	0.08	0.12	0.32	0.04	0.00	0.60	0.12	0.52
Guilt	0.08	0.08	0.04	0.00	0.12	0.00	0.24	0.08	0.00	0.20	0.16	0.32
Regret	0.04	0.00	0.08	0.00	0.12	0.04	0.28	0.04	0.04	0.40	0.16	0.40
Shame	0.28	0.00	0.04	0.08	0.04	0.00	0.20	0.12	0.12	0.16	0.12	0.36
Disappointment	0.08	0.00	0.04	0.04	0.08	0.00	0.16	0.04	0.12	0.44	0.12	0.20
Fear	0.16	0.08	0.04	0.04	0.12	0.00	0.12	0.04	0.08	0.20	0.12	0.48
Disgust	0.04	0.08	0.12	0.24	0.04	0.04	0.04	0.00	0.48	0.20	0.04	0.28
Contempt	0.08	0.04	0.08	0.04	0.04	0.00	0.08	0.00	0.24	0.36	0.04	0.40
Hate	0.52	0.04	0.08	0.00	0.00	0.00	0.00	0.04	0.04	0.24	0.04	0.48
Anger	0.72	0.12	0.12	0.00	0.00	0.00	0.12	0.04	0.04	0.04	0.04	0.48

Table S 11. Colour-emotion association matrix with the proportion of participants who associate given colours with given emotions. These proportions were derived from colour-emotion associations of non-colour-blind participants with colour terms.

	Red	Orange	Yellow	Green	Turquoise	Blue	Purple	Pink	Brown	Grey	White	Black
Interest	0.36	0.32	0.28	0.24	0.32	0.52	0.32	0.20	0.28	0.04	0.28	0.04
Amusement	0.32	0.48	0.60	0.32	0.36	0.44	0.28	0.36	0.08	0.00	0.08	0.04
Pride	0.36	0.36	0.32	0.32	0.28	0.32	0.32	0.20	0.12	0.04	0.32	0.12
Joy	0.52	0.44	0.52	0.36	0.68	0.32	0.28	0.64	0.04	0.00	0.32	0.00
Pleasure	0.68	0.56	0.48	0.40	0.56	0.48	0.28	0.72	0.08	0.00	0.32	0.00
Contentment	0.16	0.40	0.36	0.40	0.44	0.44	0.28	0.32	0.24	0.00	0.24	0.00
Admiration	0.28	0.20	0.40	0.24	0.44	0.36	0.16	0.32	0.08	0.00	0.40	0.12
Love	0.88	0.20	0.12	0.16	0.20	0.16	0.52	0.84	0.00	0.00	0.24	0.08
Relief	0.04	0.20	0.20	0.28	0.40	0.48	0.20	0.28	0.00	0.08	0.36	0.08
Compassion	0.28	0.28	0.20	0.24	0.20	0.24	0.36	0.52	0.12	0.12	0.16	0.04
Sadness	0.12	0.04	0.12	0.04	0.04	0.20	0.20	0.04	0.12	0.76	0.16	0.60
Guilt	0.28	0.08	0.20	0.04	0.00	0.04	0.08	0.00	0.08	0.36	0.08	0.40
Regret	0.24	0.04	0.16	0.16	0.00	0.12	0.16	0.16	0.20	0.56	0.08	0.44
Shame	0.24	0.12	0.28	0.08	0.00	0.00	0.20	0.04	0.28	0.32	0.00	0.44
Disappointment	0.04	0.00	0.16	0.04	0.04	0.04	0.16	0.08	0.24	0.68	0.04	0.40
Fear	0.20	0.04	0.04	0.04	0.00	0.12	0.24	0.00	0.12	0.40	0.28	0.72
Disgust	0.08	0.12	0.24	0.32	0.00	0.00	0.20	0.04	0.56	0.12	0.00	0.28
Contempt	0.16	0.04	0.16	0.24	0.00	0.08	0.16	0.04	0.28	0.32	0.04	0.36
Hate	0.60	0.00	0.08	0.04	0.00	0.00	0.00	0.04	0.04	0.20	0.16	0.52
Anger	0.76	0.12	0.04	0.00	0.00	0.04	0.08	0.00	0.04	0.16	0.04	0.40

Table S 12. Colour-emotion association matrix with the proportion of participants who associate given colours with given emotions. These proportions were derived from colour-emotion associations of colour-blind participants with colour patches.

	Red	Orange	Yellow	Green	Turquoise	Blue	Purple	Pink	Brown	Grey	White	Black
Interest	0.21	0.29	0.42	0.33	0.38	0.29	0.21	0.08	0.25	0.21	0.33	0.21
Amusement	0.21	0.42	0.50	0.25	0.33	0.29	0.08	0.38	0.04	0.08	0.13	0.04
Pride	0.25	0.21	0.29	0.21	0.33	0.17	0.17	0.13	0.08	0.08	0.25	0.08
Joy	0.17	0.46	0.63	0.33	0.46	0.50	0.13	0.46	0.04	0.08	0.21	0.04
Pleasure	0.38	0.42	0.33	0.25	0.50	0.67	0.21	0.38	0.04	0.08	0.13	0.04
Contentment	0.17	0.25	0.50	0.21	0.46	0.29	0.13	0.38	0.13	0.13	0.29	0.13
Admiration	0.13	0.33	0.42	0.17	0.33	0.42	0.13	0.38	0.13	0.13	0.25	0.08
Love	0.46	0.08	0.17	0.08	0.13	0.25	0.29	0.54	0.13	0.21	0.17	0.08
Relief	0.17	0.21	0.21	0.33	0.42	0.33	0.08	0.21	0.13	0.13	0.54	0.04
Compassion	0.21	0.17	0.25	0.13	0.21	0.29	0.33	0.33	0.17	0.17	0.25	0.04
Sadness	0.13	0.08	0.13	0.21	0.21	0.13	0.25	0.13	0.13	0.33	0.25	0.42
Guilt	0.13	0.08	0.04	0.25	0.21	0.08	0.38	0.08	0.17	0.21	0.13	0.21
Regret	0.17	0.13	0.08	0.29	0.21	0.08	0.25	0.04	0.25	0.29	0.13	0.38
Shame	0.08	0.25	0.04	0.25	0.08	0.08	0.21	0.13	0.25	0.17	0.04	0.21
Disappointment	0.08	0.13	0.08	0.25	0.17	0.08	0.21	0.13	0.21	0.38	0.17	0.50
Fear	0.25	0.04	0.13	0.08	0.08	0.13	0.13	0.13	0.08	0.13	0.17	0.25
Disgust	0.17	0.13	0.08	0.33	0.08	0.08	0.13	0.13	0.71	0.21	0.13	0.33
Contempt	0.13	0.08	0.13	0.25	0.08	0.08	0.21	0.08	0.38	0.25	0.04	0.33
Hate	0.38	0.08	0.08	0.04	0.04	0.04	0.17	0.13	0.25	0.13	0.04	0.17
Anger	0.54	0.17	0.08	0.08	0.04	0.04	0.08	0.04	0.17	0.08	0.04	0.17

Table S 13. Colour-emotion association matrix with the proportion of participants who associate given colours with given emotions. These proportions were derived from colour-emotion associations of non-colour-blind participants with colour patches.

	Red	Orange	Yellow	Green	Turquoise	Blue	Purple	Pink	Brown	Grey	White	Black
Interest	0.06	0.06	0.26	0.55	0.32	0.48	0.10	0.13	0.19	0.10	0.23	0.10
Amusement	0.19	0.35	0.48	0.19	0.48	0.32	0.26	0.48	0.00	0.03	0.06	0.00
Pride	0.26	0.29	0.26	0.23	0.26	0.29	0.16	0.03	0.06	0.06	0.06	0.03
Joy	0.19	0.52	0.55	0.35	0.58	0.42	0.26	0.42	0.06	0.06	0.16	0.03
Pleasure	0.39	0.35	0.42	0.42	0.52	0.48	0.26	0.61	0.13	0.06	0.10	0.00
Contentment	0.03	0.35	0.29	0.48	0.48	0.42	0.29	0.23	0.16	0.13	0.26	0.03
Admiration	0.23	0.29	0.29	0.26	0.29	0.35	0.23	0.23	0.10	0.03	0.19	0.03
Love	0.55	0.16	0.06	0.13	0.19	0.19	0.32	0.74	0.00	0.03	0.03	0.00
Relief	0.06	0.16	0.13	0.35	0.32	0.42	0.26	0.26	0.16	0.23	0.55	0.10
Compassion	0.16	0.26	0.10	0.16	0.13	0.26	0.29	0.45	0.10	0.10	0.16	0.06
Sadness	0.06	0.03	0.06	0.10	0.03	0.16	0.16	0.06	0.16	0.45	0.16	0.35
Guilt	0.19	0.13	0.10	0.13	0.06	0.06	0.13	0.10	0.16	0.23	0.03	0.32
Regret	0.13	0.03	0.03	0.13	0.16	0.16	0.13	0.13	0.29	0.39	0.23	0.32
Shame	0.19	0.06	0.00	0.06	0.00	0.00	0.13	0.03	0.16	0.16	0.03	0.13
Disappointment	0.06	0.03	0.03	0.10	0.03	0.03	0.10	0.03	0.32	0.39	0.16	0.42
Fear	0.19	0.10	0.10	0.00	0.00	0.03	0.03	0.03	0.03	0.26	0.10	0.35
Disgust	0.23	0.03	0.13	0.26	0.00	0.00	0.13	0.03	0.52	0.13	0.03	0.55
Contempt	0.32	0.10	0.16	0.03	0.00	0.00	0.13	0.03	0.23	0.10	0.00	0.52
Hate	0.61	0.06	0.10	0.03	0.00	0.00	0.00	0.00	0.10	0.03	0.00	0.16
Anger	0.68	0.13	0.10	0.06	0.00	0.00	0.03	0.06	0.03	0.03	0.06	0.13

Table S 14. Confusion matrix for the colour-naming task. The columns represent colour patches that were given to the self-reported colour-blind participants (*n* = 22). The rows represent colour terms given to participants. The numbers represent mean evaluation of the likelihood that each colour patch would be named with each colour term (0-100). The values in bold mark the most likely colour term per colour patch.

		Presented colour patch											
		Black	Blue	Brown	Green	Grey	Orange	Pink	Purple	Red	Turquoise	White	Yellow
Evaluated colour term	Black	25.03	0.25	1.04	0.33	0.39	0.22	0.42	0.43	0.23	0.49	0.18	0.06
	Blue	1.10	**93.78**	0.21	0.20	15.53	0.16	9.48	21.74	0.35	**86.14**	1.19	0.20
	Brown	44.83	0.04	**82.31**	6.86	0.22	5.77	0.10	0.06	5.70	0.30	0.12	0.23
	Green	**71.32**	0.11	32.48	**91.85**	8.78	4.15	1.34	0.46	1.22	13.61	0.24	5.58
	Grey	20.61	0.95	1.85	0.41	**90.13**	0.04	28.00	3.44	0.21	15.69	7.70	0.17
	Orange	0.40	0.20	10.90	4.17	0.16	**94.65**	0.42	0.43	12.04	0.19	0.35	19.34
	Pink	0.82	7.54	0.12	0.13	33.01	2.01	**91.31**	50.78	26.38	8.83	6.73	0.08
	Purple	0.05	13.98	0.44	0.25	4.39	0.15	9.68	**81.00**	3.28	9.86	0.17	0.10
	Red	1.71	0.28	10.98	0.18	2.47	11.86	5.99	5.98	**93.98**	0.82	0.41	1.58
	Turquoise	3.99	49.02	1.12	0.92	26.98	0.37	11.70	21.51	4.30	65.37	0.73	1.08
	White	0.25	0.93	0.05	0.18	10.17	0.13	10.71	0.25	0.17	5.51	**94.51**	0.06
	Yellow	0.11	0.34	4.02	4.94	0.53	21.25	0.16	0.08	1.49	0.15	0.98	**93.53**

Table S 15. Confusion matrix for the colour-naming task. The columns represent colour patches that were given to the self-reported non-colour-blind participants (*n* = 33). The rows represent colour terms give to participants. The numbers represent mean evaluation of the likelihood that each colour patch would be named with each colour term (0-100). The values in bold mark the most likely colour term per colour patch; several values are marked if they have similar likelihood.

		Presented colour patch											
		Black	Blue	Brown	Green	Grey	Orange	Pink	Purple	Red	Turquoise	White	Yellow
Evaluated colour term	Black	**49.97**	0.27	1.01	0.05	3.03	0.05	0.43	0.20	0.11	0.20	0.12	0.19
	Blue	2.32	**97.17**	0.48	1.36	9.73	0.57	0.09	0.84	0.16	**92.41**	0.11	1.33
	Brown	12.04	0.61	**96.55**	0.42	3.31	5.94	0.52	0.78	0.47	0.05	0.14	0.55
	Green	**38.54**	1.50	1.28	**99.29**	1.13	0.58	0.05	0.26	0.04	2.79	0.04	0.15
	Grey	**45.31**	1.10	0.56	1.13	**97.42**	0.07	0.11	0.64	0.29	0.80	9.73	0.42
	Orange	0.05	0.29	12.45	0.12	0.10	**92.65**	0.49	0.12	10.22	0.16	0.21	3.73
	Pink	0.18	0.34	0.10	0.67	0.37	1.13	**98.88**	10.42	18.07	1.69	0.53	0.58
	Purple	0.63	1.76	0.62	0.11	1.56	0.19	15.55	**99.24**	0.67	0.27	0.29	0.43
	Red	0.63	0.08	3.24	0.38	0.08	3.26	10.28	3.47	**97.26**	0.86	0.13	1.57
	Turquoise	2.58	54.46	0.23	1.77	2.72	0.51	2.15	3.66	0.19	**82.57**	0.20	0.03
	White	0.04	1.19	0.48	0.04	16.81	0.08	2.85	0.06	0.24	1.03	**99.69**	0.19
	Yellow	0.28	0.42	6.41	0.58	0.34	25.59	0.32	0.13	0.64	0.26	0.27	**99.89**
